# Evaluation of *in vitro* toxicity of common phytochemicals included in weight loss supplements using ^1^H NMR spectroscopy

**DOI:** 10.1002/2211-5463.70170

**Published:** 2025-12-09

**Authors:** Emily C. Davies, Garth L. Maker, Ian F. Musgrave, Samantha Lodge

**Affiliations:** ^1^ Centre for Computational and Systems Medicine Murdoch University Perth Australia; ^2^ Medical, Molecular and Forensic Sciences Murdoch University Australia; ^3^ Adelaide Medical School The University of Adelaide Australia

**Keywords:** ^1^H NMR spectroscopy, Caco‐2 cells, epigallocatechin‐3,0‐gallate, HepG2 cells, herbal weight loss supplements, oxidative stress

## Abstract

Herbal and dietary supplements (HDS) are popular among consumers seeking a ‘natural’ approach for improving their health; however, at present, there is a lack of evidence to support the claims of efficacy and safety for most of these products. Herbal weight loss supplements (WLS) are a group of HDS that are frequently implicated in cases of toxicity; however, the causative substances often remain unknown due to the complex chemical nature of such supplements. This study aimed to analyse the *in vitro* safety (in human liver carcinoma (HepG2) cells and colon carcinoma (Caco‐2) cells) of 12 active compounds commonly found in WLS, first with safety screening using the MTT cytotoxicity assay, followed by metabolic profiling with ^1^H NMR spectroscopy. Of the phytochemicals evaluated, epigallocatechin‐3,0‐gallate (EGCG) was the only compound that caused a significant reduction in the viability of both cell lines (25.3% in HepG2 cells and 18.5% in Caco‐2 cells), and this decrease was potentiated by CYP450 induction with rifampicin. Subsequent ^1^H NMR analysis showed changes in key metabolites such as amines, amino acids, carboxylic acids, and glucose that were indicative of protein degradation and disrupted energy and lipid metabolism. While the remaining 11 active compounds analysed did not demonstrate significant toxicity in isolation, these require further assessment to determine their safety when used in combination with other phytochemicals. Given that the majority of WLS contain multiple herbal ingredients, each with a complex chemical composition, it is important to understand the role of interactions in adverse events.

## Graphical abstract

We investigated the toxicity of 12 active compounds commonly found in herbal weight loss supplements (WLS) using human liver and colon cell models. Epigallocatechin‐3‐gallate was the only compound showing significant toxicity. Metabolic profiling revealed protein degradation, disrupted energy and lipid metabolism suggesting that the inclusion of EGCG in WLS should be more stringently regulated to ensure consumer safety.

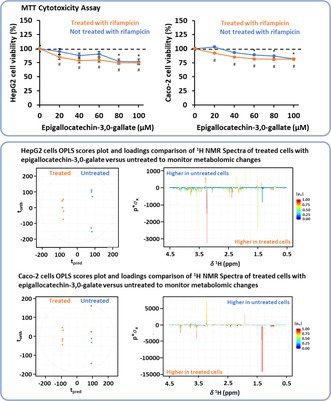

**Abbreviations**
ANOVA: analysis of variance
ARTG: Australian Register of Therapeutic Goods
AUROC: area under the receiver operator curve
BBI: broadband decoupling inverse
Caco‐2: colon carcinoma
CYP3A: cytochrome P450 3A
CYP450: cytochrome P450
D_2_O: Deuterium oxide
DILI: drug‐induced liver injury
DMEM: Dulbecco's Modified Eagle Medium
DMSO: Dimethyl sulfoxide
EDTA: Ethylenediaminetetraacetic acid
EGCG: Epigallocatechin‐3,0‐gallate
FBS: fetal bovine serum
fc: fold change
FDA: Food and Drug Administration
GTE: green tea extract
HCA: hydroxycitric acid
HDS: herbal and dietary supplements
HepG2: human liver carcinoma
HFP: human Foods Program
HILI: herb‐induced liver injury
MeOH: Methanol
MTT: Thiazolyl blue tetrazolium bromide
NMR: Nuclear Magnetic Resonance
OPLS‐DA: orthogonal projection to latent structures discriminant analysis
PBS: Phosphate buffered saline
PCA: Principal component analysis
PQN: probabilistic quotient normalisation
R2X: model interpretation rate
ROS: reactive oxygen species
SEM: standard error of the mean
SSRIs: selective serotonin reuptake inhibitors
TCA: Tricarboxylic acid
TGA: Therapeutic Goods Administration
WHO: World Health Organisation
WLS: weight loss supplements

Herbal and dietary supplements (HDS) are a popular group of complementary medicines that contain naturally derived ingredients and are available for purchase off‐the‐shelf without medical supervision [1]. The World Health Organization (WHO) estimates that 85% of developing countries use herbal supplements as part of their health care [2]. While dietary supplements are generally marketed to replace missing nutrients in the diet, herbal supplements often have additional purported health benefits surrounding the protection against and treatment of certain conditions such as cardiovascular disease, inflammation and obesity [1]. Despite these claims, there is little evidence available to support the efficacy of these products for these purposes [3].

Current regulation governing the use of HDS is vastly different worldwide. In the United States, the Food and Drug Administration (FDA) has guidance documents related to dietary supplements [4], and they are regulated under the Dietary Supplement Health and Education Act of 1994. The regulation of HDS is less stringent than that for pharmaceuticals and does not require FDA approval before marketing. Indeed, it is the manufacturers who are responsible for the accurate labelling and safety of their products. However, in 2024, the FDA started an initiative, the Human Foods Program (HFP) to enhance regulatory efficiency focusing on consumer safety, requiring manufacturers to submit evidence of safety before marketing [5]. In Australia, their use is governed by the Therapeutic Goods Administration (TGA) where HDS are listed as complementary medicines, and most are listed on the Australian Register of Therapeutic Goods (ARTG) [6].

The regulation of HDS is typically less stringent than for other medicinal products such as prescription drugs and over‐the‐counter medicines, with independent review of supplement efficacy, composition and safety predominantly limited to randomised postmarket analysis [7].

In recent years, the combination of widespread HDS use and insufficient regulation of these products has raised concerns about consumer safety. HDS have been increasingly associated with adverse events, despite the widespread belief among consumers that their natural ingredients make them safe [8, 9, 10]. Adverse events have been reported impacting the gastrointestinal, cardiovascular, renal and central nervous systems [11, 12, 13, 14, 15, 16, 17, 18]. While many studies have investigated individual medicinal plants, a recent review demonstrated significant adverse effects were also found in herbal formulas that composed two or more herbs [19]. It was found that while the symptoms included nausea, vomiting and diarrhoea, the most frequently reported organ system was the hepatobiliary, then nervous, followed by gastrointestinal [19]. A further study aimed to investigate the reporting rates of adverse events due to herbal medicines and found that reporting rates varied considerably while only the main ingredients were disclosed, while other potentially harmful ingredients were not listed [20]. The liver is particularly susceptible to toxicity, given its role in the metabolism of xenobiotics administered orally [8]. The term herb‐induced liver injury (HILI) is currently used to describe liver dysfunction caused by herbal medicines and supplements [9, 21, 22, 23] and the condition continues to increase in prevalence each year [24]. HILI is a major health concern that is complicated by underreporting of HDS use to medical practitioners [23].

Weight loss supplements (WLS) are a popular group of HDS, which have seen a steady rise in usage in recent years [24] with their global retail value surpassing US$5 billion [25]. These products are commonly sold in pharmacies, health food stores, supplement outlets and online and are therefore readily accessible. It is the combination of this accessibility, perceived safety and the ongoing obesity epidemic that is thought to be responsible for the popularity of these supplements. The increased use of WLS has been associated with a growing number of HILI cases in relation to these products [14, 26, 27, 28, 29]. Hydroxycut, a popular brand of WLS with many different formulations, has been implicated in over 50 cases of hepatotoxicity [26, 27, 28]. Some Hydroxycut products have been removed from the market due to safety concerns; however, these often reappear on the market with a different name. Exolise is another weight loss product that has been implicated in numerous adverse events, at least one of which required liver transplantation, and has since been removed from the market [27, 29]. In most cases, the causative herb or phytochemical is unclear; however, certain herbal ingredients such as *Garcinia gummi‐gutta* (L.) N.Robson (*Clusiaceae*) (also referred to as *Garcinia cambogia*) and *Camellia sinensis* (L.) Kuntz (*Theaceae*) have been identified as potentially problematic [14, 30, 31].

Herbs included in supplements are compositionally complex, comprised of multiple phytochemicals with differing biological activities. Examples of herbs commonly included in WLS include *C. sinensis*, *G. gummi‐gutta*, *Coffea arabica* L. (*Rubiaceae*), *Coffea canephora* Pierre ex A.Froehner (*Rubiaceae*), *Citrus aurantium* L. (*Rutaceae*), *Capsicum annuum* (L.) (*Solanaceae*), *Coleus barbatus* (Andrews) Benth. ex G.Don (*Lamiaceae*) and *Rubus idaeus* L. (*Rosaceae*) [18]. Phytochemicals in these herbs that are purportedly beneficial for weight management include green tea catechins, hydroxycitric acid, chlorogenic acid, synephrine, capsaicin, forskolin and raspberry ketone; however, claims that these compounds can induce a clinically significant and sustainable reduction in body weight remain unsubstantiated [18]. There is also little safety data available for many of these phytochemicals, including how these compounds interact with each other when used in combination. A further consideration is the effect of herbal weight loss supplements when used in conjunction with prescribed medication. A recent study showed that drug‐induced liver injury (DILI) correlated with turmeric supplementation and the concurrent use of semaglutide, a prescribed medication for weight loss. While cessation of the herbal supplement improved liver function [32].

Given the prevalence of adverse events associated with WLS, it is important to improve current understanding of the phytochemicals included in these products to better predict problematic ingredients and herbal combinations. The aim of this study was to examine 12 phytochemicals found in seven herbal ingredients commonly included in WLS to determine their toxicity in human intestinal and hepatic cells. Phytochemicals that caused significant cytotoxicity in one or both cell lines were analysed using ^1^H NMR spectroscopy to characterise the metabolic changes that occur in response to these compounds.

## Methods

### Toxicological screening

#### Active compound preparations

Twelve active compounds commonly found in herbal WLS were selected for toxicological screening: epigallocatechin‐3,0‐gallate (EGCG), epigallocatechin, epicatechin, catechin hydrate, chlorogenic acid, synephrine, raspberry ketone, capsaicin, forskolin, caffeine anhydrous, hydroxycitric acid lactone and hydroxycitric acid tripotassium salt (Table 1). Each compound was dissolved in DMSO and subsequently diluted in serum‐free DMEM to create a 1 mm stock solution with 10% DMSO. Stock solutions were diluted with serum‐free DMEM to obtain final treatment concentrations of 20, 40, 60, 80 and 100 μM with 1% DMSO. A vehicle control of serum‐free DMEM containing 1% DMSO was also prepared, to match the final DMSO concentration in the treatments. The range of concentrations selected was based on those used in similar studies examining the cytotoxicity of herbal active compounds [33, 34, 35] and agrees with concentrations used in recent studies [36].

**Table 1:** Primary active compounds found in herbal ingredients commonly included in WLS.

Herbal ingredient	Active compound(s) analysed
*Camellia sinensis*	Epigallocatechin‐3,0‐gallate, epigallocatechin, epicatechin, catechin hydrate, caffeine
*Coffea* spp.	Chlorogenic acid, caffeine
*Citrus aurantium*	Synephrine
*Rubus idaeus*	Raspberry ketone
*Capsicum annuum*	Capsaicin
*Plectranthus barbatus*	Forskolin
*Garcinia gummi‐gutta*	Hydroxycitric acid (lactone, tripotassium salt)

#### Cell culture

Due to adverse events associated with WLS commonly involving the gastrointestinal system, human liver carcinoma cells (HepG2, accession number: CVCL_0027) and colon carcinoma cells (Caco‐2, accession number: CVCL_0025) were selected for toxicological analysis. Both cell lines were cultured in 75‐cm^2^ flasks and grown in supplemented Dulbecco's Modified Eagle Medium (DMEM) and incubated at 37°C and 5% carbon dioxide. Supplemented DMEM contained 10% fetal bovine serum (FBS), 1% nonessential amino acids and 1% penicillin–streptomycin. HepG2 cells were passaged every 6–7 days and Caco‐2 cells every 4–5 days, with 0.5% trypsin–EDTA used to detach cells from flasks. Cell counts were performed manually using the Trypan blue exclusion method. Ninety‐six‐well plates were used for cell viability experiments, with a seeding density of 1.2 × 10^4^ cells per well. Cells for ^1^H‐NMR spectroscopy were grown in six‐well plates, seeded at a density of 1.0 × 10^6^ cells per well. Prior to experiments commencing, all plates were incubated for 48 h to allow cells to equilibrate and adhere to the wells.

#### Bioactive pretreatment

The majority of drug and phytochemical metabolism is catalysed by the cytochrome P450 3A (CYP3A) enzyme family with CYP3A4 being the most common isoform. CYP450 activity in HepG2 cells has been shown to be significantly lower than observed in hepatocytes and intestinal epithelial cells *in vivo* [37, 38]. Given that metabolism of phytochemicals can enhance their toxicity via the production of toxic metabolites, it was important to initially assess phytochemical toxicity in the presence and absence of CYP450 induction. As rifampicin has been demonstrated to induce the activity of many CYP450 enzymes including CYP3A [39, 40] was added to supplemented DMEM at a concentration of 2 mm for 48 h prior to WLS exposure.

#### Cell viability

Thiazolyl blue tetrazolium bromide (MTT) cytotoxicity assay was used to analyse cell viability following exposure to WLS for 48 h. Viable cells take up MTT and convert it to an insoluble blue formazan product. Colour intensity is therefore directly proportional to mitochondrial activity in living cells. Both cell lines were incubated with WLS dilutions, including control, for 48 h. At the conclusion of the exposure period, all treated media were aspirated and replaced with serum‐free medium containing 0.25 mgmL^−1^ MTT. Plates were incubated for 3 h; MTT solution was aspirated and replaced with DMSO to lyse cells. A Tecan Spark Microplate Reader was subsequently used to measure absorbance at 570 nm.

#### Statistical analysis

Statistical analysis of MTT data was completed using IBM SPSS Statistics (v29.0.1.0). MTT data were first investigated using a one‐way analysis of variance (ANOVA) to determine the effects of WLS compared to the control. This was followed by a Dunnett's *post hoc* test to determine *P‐*values at each concentration compared to control, with a significance of *P* < 0.01 selected for all experiments. Further data analysis and generation of cell viability graphs were carried out in Microsoft Excel (v16.75.2). Graphs depict the mean ±SEM at each concentration, with significance shown as *P < 0.01.

### NMR toxicological analysis

#### Active compound preparations and cell culture

Active compounds that significantly decreased cell viability were selected for further toxicological analysis. All treatment groups, including vehicle controls, were prepared as outlined for MTT assays, as were the HepG2 and Caco‐2 cells. Six‐well plates were used to prepare cells for ^1^H NMR spectroscopy experiments, and cells were seeded at a density of 1.0 × 10^6^ cells per well. In order to best emulate *in vivo* responses, all cells were pretreated with rifampicin for 48 h to induce CYP3A activity prior to experimentation. Following 48 h exposure, spent medium was removed and cells were washed with PBS before being harvested into MeOH. Samples were lysed using a Bertin Technologies Precellys 24 tissue lyser at 6500 rpm for 2 × 20 s and subsequently centrifuged at 16 100 ***g*** for 5 min, with the supernatant transferred to fresh tubes and dried for storage at −80 °C.

#### ^1^H NMR spectroscopy data acquisition and processing parameters

All ^1^H NMR spectroscopy was carried out on a 600 MHz Bruker Avance III HD spectrometer equipped with a 5‐mm BBI probe, which underwent quantitative calibration prior to analysis using a previously described protocol [41]. Cell extracts were thawed and resuspended in 540 μL D_2_O and 60 μL phosphate buffer (1.5 m KH_2_PO_4_, 2 mm NaN_3_, 0.1% TSP, pH 7.4) and transferred to a 5‐mm Bruker (Bruker Biosoin GmbH, Rheinstetten 76287, Germany) SampleJet NMR tube, which was closed with caps and sealed with POM balls. All NMR experiments were completed at 300 K. For each sample, a one‐dimensional (1D) ^1^H experiment (256 scans, 65 536 data points, acquisition time of 2.73 s, and a spectral width of 12019.23 Hz/20 ppm) was completed in automation mode, amounting to a total of 32‐min acquisition time per sample. All data were processed in automation using Bruker TopSpin™ v3.6.2 and ICON™ NMR to achieve phasing and baseline correction.

#### NMR data modelling

The statistical programming language R (v4.1.3) and the metabom8 package (v1.0.0) were used for all data analysis (https://tkimhofer.github.io/metabom8). To prepare spectral datasets for each WLS group for multivariate modelling, spectral regions corresponding to residual water resonance signal (δ 4.60–4.85), methanol resonance signal (δ 3.27–3.35) and noise (δ < 0.5 and δ > 9.5) were excluded from analyses, and baseline was corrected using an asymmetric least squares routine for each spectrum. Spectra were normalised using a probabilistic quotient normalisation (PQN) method with the median spectrum as reference, and data were mean‐centred and scaled to unit variance prior to multivariate modelling [42]. Principal component analysis (PCA) was performed to determine key sources of structural variation within each dataset. To identify metabolites primarily responsible for changes observed in WLS treated cells, a previously constructed orthogonal projection to latent structures discriminant analysis (OPLS‐DA) model was used [43]. This was performed for 1D spectral datasets for each WLS group separately, with the optimal number of orthogonal components selected using the area under the receiver operator curve (AUROC) calculated from predictive component scores. Metabolite fold changes were determined by cutting integral regions of the metabolite peaks in the spectrum and comparing these between treated and untreated HepG2 and Caco‐2 cells. Fold changes greater than 1 indicate an increase in the metabolite with treatment, while those below 1 signify a decrease in the metabolite with treatment. IBM SPSS Statistics (v29.0.1.0) was used to determine statistical significance of fold changes, denoted as *P < 0.01.

## Results

### Toxicological screening

**Fig. 1:**
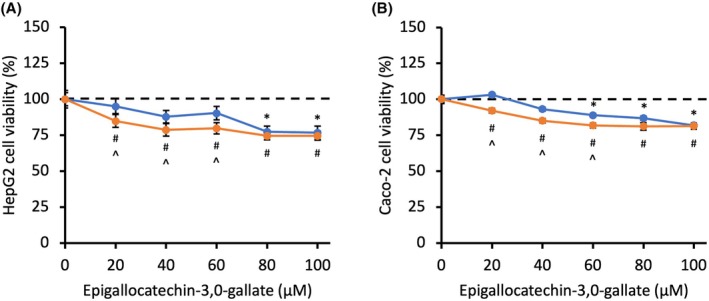
HepG2 (left) and Caco‐2 (right) cell viability following treatment with EGCG for 48 h (*n* = 15, *p* < 0.01). Orange: cells pretreated with rifampicin to induce CYP450 activity; blue: cells did not undergo pretreatment. */^#^ indicate concentrations where cell viability is significantly different from cells not treated with EGCG. ^ denotes a significant difference between rifampicin‐induced and non‐induced cells. MTT data were first investigated using a one‐way analysis of variance (ANOVA) to determine the effects of WLS compared to the control. This was followed by a Dunnett's *post hoc* test to determine *p*‐values at each concentration compared to control.

EGCG was the only active compound that caused significant toxicity in HepG2 and Caco‐2 cells, and induction with rifampicin appeared to enhance this (*P* < 0.01) (Fig. 1, Figs S1–S11). In both cell lines pre‐treated with rifampicin, a concentration of 100 μm was selected for ^1^H NMR spectroscopy as it reduced cell viability by 25.3% in HepG2 cells and 18.5% in Caco‐2 cells. The remaining 11 compounds tested did not cause a significant reduction in cell viability; however, all yielded a significant increase in cell viability in one or both cell lines in response to at least one concentration of these phytochemicals (Figs S1–S11). Both cell lines had an increase in cell viability when exposed to epicatechin, catechin hydrate, synephrine, capsaicin, forskolin, caffeine and hydroxycitric acid tripotassium salt. An increase in cell viability was also observed in Caco‐2 cells in response to epigallocatechin, chlorogenic acid and hydroxycitric acid lactone, and in HepG2 cells treated with raspberry ketone. A significant difference in response to the phytochemicals was observed between rifampicin‐induced and noninduced cells at varying concentrations for all except catechin hydrate, raspberry ketone and hydroxycitric acid lactone (Fig. 1, Figs S1–S11).

### NMR analysis of *in vitro* toxicity

**Fig. 2:**
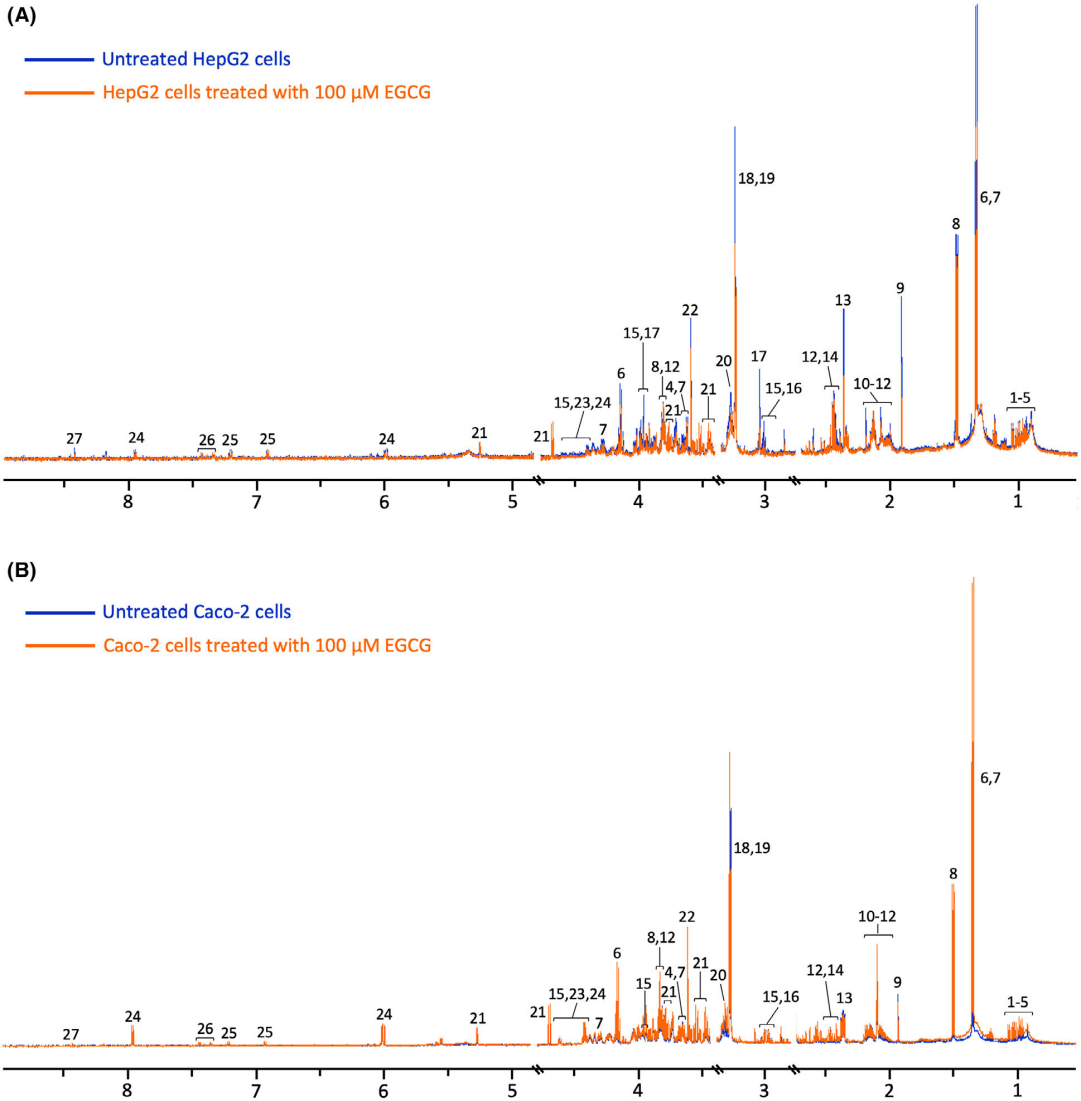
^1^H NMR spectra of (A) HepG2 and (B) Caco‐2 cell extracts, comparing untreated (blue) and EGCG‐treated (orange) groups. (1) 3‐methyl‐2‐oxovalerate, (2) 2‐oxoisocaproate, (3) leucine, (4) valine, (5) isoleucine, (6) lactate, (7) threonine, (8) alanine, (9) acetate, (10) proline, (11) glutamate, (12) glutamine, (13) pyruvate, (14) succinate, (15) glutathione, (16) lysine, (17) creatine, (18) phosphocholine, (19) glycerophosphocholine, (20) betaine, (21) glucose, (22) glycine, (23) NAD+, (24) UDP‐glucose, (25) tyrosine, (26) phenylalanine, (27) formate.

Representative ^1^H NMR spectra of EGCG‐treated and untreated HepG2 and Caco‐2 cell extracts are depicted in Fig. 2. Acetate, citrate, formate, glucose, isoleucine, lactate, phosphocholine, threonine and valine were among 20 key metabolites identified in ^1^H NMR spectra of HepG2 (Fig. 2A) and 30 in Caco‐2 cells (Fig. 2B).

**Fig. 3:**
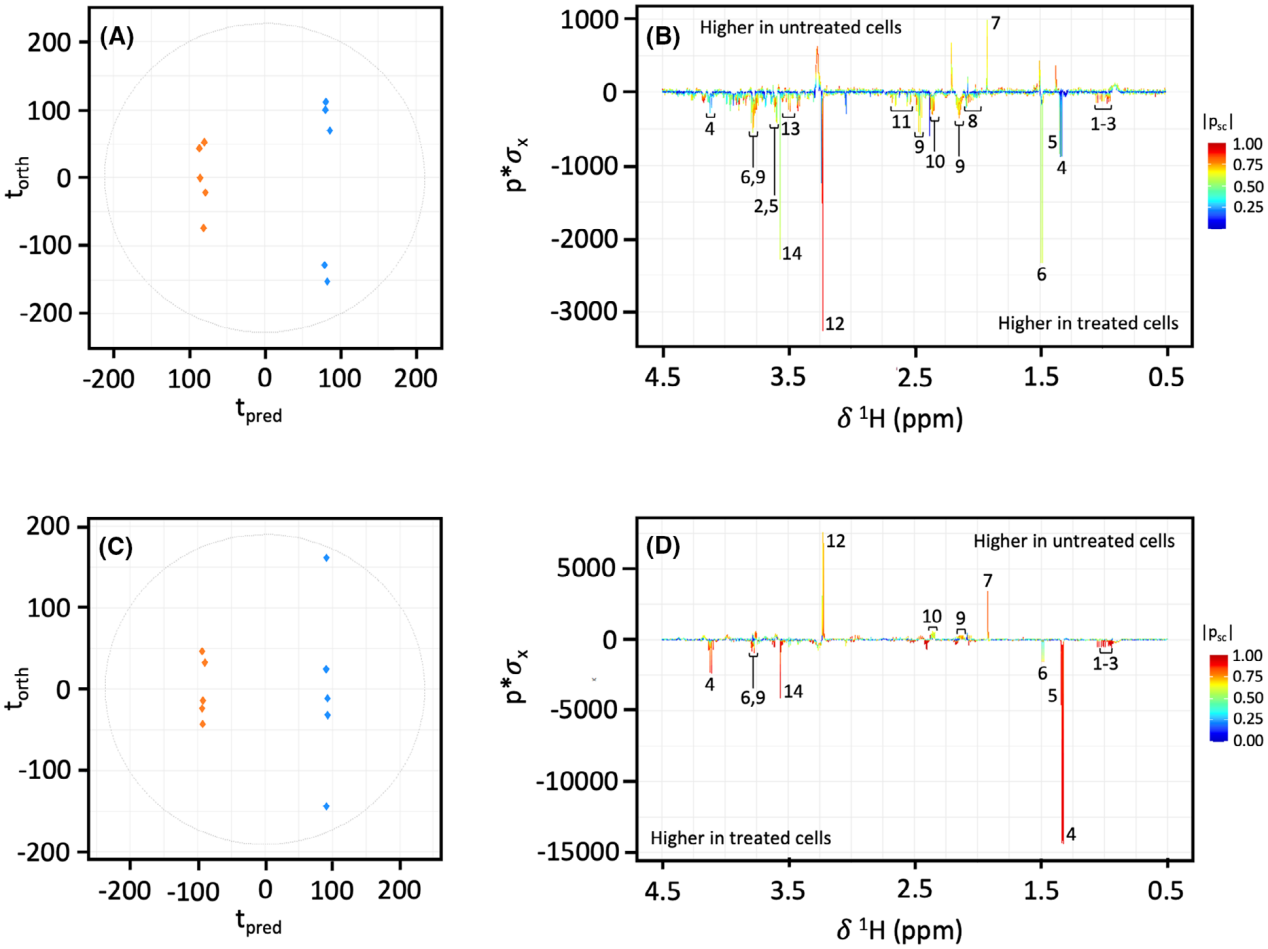
OPLS‐DA scores plots (CV‐AUROC = 1.0) and corresponding coefficient loadings plots derived from ^1^H NMR spectra of HepG2 (A, B) and Caco‐2 cells (C, D) in untreated (blue) and EGCG‐treated (orange) groups. (1) leucine, (2) valine, (3) isoleucine, (4) lactate, (5) threonine, (6) alanine, (7) acetate, (8) proline, (9) glutamine, (10) glutamate, (11) citrate, (12) phosphocholine, (13) glucose, (14) glycine.

OPLS‐DA comparing NMR spectra of HepG2 and Caco‐2 cells treated with 100 μm EGCG to untreated controls showed a clear difference in metabolite profile (Fig. 3A,C). The R2X values for each model were 0.16 (Fig. 3A) and 0.19 (Fig. 3C), and AUROC and CV‐AUROC were 1.0 for both, indicating goodness of fit and good predictability. Corresponding OPLS‐DA coefficient loadings plots demonstrate key metabolites contributing significantly to the observed variation between treated and untreated HepG2 and Caco‐2 cells (Fig. 3B,D).

A complete overview of metabolite fold changes and corresponding ^1^H and ^13^C chemical shifts in HepG2 and Caco‐2 cells treated with EGCG is outlined in Table 2. Treated HepG2 cells had a significant reduction in acetate (fc = 0.84) and formate (fc = 0.81) compared to untreated controls (Table 2). Most metabolites were increased compared to untreated HepG2 cells; those that were significantly increased included alanine (fc = 1.33), citrate (fc = 1.47), glutamate (fc = 1.25), glycine (fc = 1.58), leucine (fc = 1.16), phosphocholine (fc = 1.75), proline (fc = 1.48) and threonine (fc = 1.49). Caco‐2 cells treated with EGCG had significantly reduced levels of acetate (fc = 0.59), formate (fc = 0.85), glutamine (fc = 0.85), phosphocholine (fc = 0.71) and pyruvate (fc = 0.63). Increased levels of alanine (fc = 1.10), aspartate (fc = 1.29), creatine (fc = 1.49), glycine (fc = 1.52), isoleucine (fc = 1.36), lactate (fc = 1.46), leucine (fc = 1.29), myo‐inositol (fc = 1.47), pyroglutamate (fc = 1.47) and valine (fc = 1.47) were observed (Table 2).

**Table 2:** Metabolite changes in HepG2 cells identified via ^1^H NMR analysis of cell extracts following treatment with 100 μm EGCG for 48 h. Fold changes indicate metabolite concentration in treated HepG2 relative to untreated cells, with values < 1.0 signifying a decrease and > 1.0 an increase. Statistically significant differences in metabolite levels in treated cells compared to untreated are denoted by *P < 0.05 and **P < 0.01.

Metabolites	δ ^1^H ppm and multiplicity	δ ^13^C ppm	Fold change	
			HepG2	Caco‐2
Acetate	1.92 (s)	26.10	0.84*	0.59**
Alanine	1.49 (d), 3.79 (q)	17.21, 54.60	1.33**	1.10*
Asparagine	2.88 (dd), 2.98 (dd), 3.99 (t)	35.70, 52.40, 174.10, 175.30	—	1.12
Aspartate	2.68 (dd), 2.80 (dd), 3.90 (t)	39.30, 55.09	—	1.12
Citrate	2.54 (d), 2.65 (d)	48.70, 48.70	1.47*	0.93
Creatine	3.04 (s), 3.93 (s)	40.11, 56.23	—	1.49*
Formate	8.43 (s)	172.11	0.81*	0.85**
Fumarate	6.52 (s)	132.45, 166.73	1.39	—
Glucose	3.25 (dd), 3.41 (t), 3.42 (t), 3.47 (m), 3.50 (t), 3.54 (dd), 3.72 (t), 3.73 (dd), 3.77 (dd), 3.83 (m), 3.84 (m), 3.90 (dd), 4.65 (d), 5.24 (d)	77.02, 73.50, 78.65, 63.53, 74.15, 63.32, 63.39, 96.00	1.14	1.07
Glutamate	2.05 (m), 2.14 (m), 2.34 (m), 2.37 (m), 3.76 (q)	29.70, 36.24, 57.57	1.25**	0.88
Glutamine	2.14 (m), 2.46 (m), 3.78 (t)	29.51, 32.94, 57.10	1.24	0.85**
Glutathione	2.16 (m), 2.19 (m), 2.54 (m), 2.57 (m), 2.93 (dd), 2.98 (dd), 3.76 (dd), 3.77 (t), 3.80 (dd), 4.57 (dd), 8.26 (m), 8.51 (s)	29.06, 34.05, 28.32, 46.35, 57.17, 59.21, 58.55	—	0.90
Glycerophosphocholine	3.23 (s), 3.69 (m), 3.92 (m), 4.32 (dd)	55.90, 68.10, 73.90, 64.40	—	0.93
Glycine	3.56 (s)	44.51	1.58**	1.52**
Isoleucine	0.96 (t), 1.02 (d), 1.47 (m)	13.91, 17.37, 27.00	1.10	1.36**
Lactate	1.33 (d), 4.11 (q)	20.20, 68.60	1.09	1.46**
Leucine	0.96 (d), 0.97 (d), 1.68 (m), 1.71 (m), 1.73 (m), 3.74 (dd)	23.40, 24.30, 42.60, 56.90	1.16**	1.29**
Lysine	1.44 (m), 1.48 (m), 1.72 (m), 1.88 (m), 1.92 (m), 3.02 (t), 3.77 (t)	24.04, 29.15, 42.12, 57.45	—	0.97
Methylmalonate	1.22 (d), 3.17 (q)	17.91	—	1.16
Myoinositol	3.28 (t), 3.54 (dd), 3.63 (t), 4.07 (t)	75.24	—	1.47**
Phenylalanine	3.13 (dd), 3.29 (dd), 3.98 (dd), 7.34 (m), 7.38 (m), 7.43 (m)	38.90, 38.90, 58.90, 132.20, 129.80, 131.90	—	1.06
Phosphocholine	3.22 (s), 3.60 (m), 4.15 (m)	56.52, 68.90, 60.61	1.75**	0.71*
Proline	1.99 (m), 2.03 (m), 2.07 (m), 2.37 (m), 3.34 (m), 3.42 (m), 4.13 (dd)	24.84, 30.13, 47.42, 62.29, 175.27	1.48*	0.80
Pyroglutamate	2.03 (m), 2.39 (m), 2.42 (m), 2.50 (m), 4.17 (dd)	32.28, 27.98, 60.97	—	1.47**
Pyruvate	2.38 (s)	29.50	—	0.63**
Serine	3.85 (dd), 3.96 (dd), 3.99 (dd)	57.40, 61.31, 173.37	1.17	—
Succinate	2.41 (s)	37.30	—	0.99
Threonine	1.34 (d), 3.59 (d), 4.26 (m)	22.30, 63.46	1.49*	0.80
Tyrosine	3.06 (dd), 3.20 (dd), 3.97 (dd), 6.91 (m), 7.20 (m)	37.90, 59.40, 118.80, 133.40	1.03	1.06
UDP‐glucose	3.47 (t), 3.55 (m), 3.77 (t), 3.79 (dd), 3.86 (dd), 3.89 (m), 4.20 (m), 4.24 (m), 4.29 (m), 4.36 (m), 4.38 (m), 5.60 (dd), 5.98 (d), 5.99 (d), 7.96 (d)	60.08, 64.69, 68.94, 71.33, 73.52, 82.96, 88.19, 95.31, 102.36, 141.18, 151.26, 164.21	1.08	0.99
Valine	0.99 (d), 1.05 (d), 2.28 (m), 3.61 (d)	19.41, 20.75, 31.89	1.10	1.47**

## Discussion

The use of herbal and dietary supplements has been on the rise in recent years and of these, WLS have become increasingly popular [24]. Suggested reasons for this include ease of access, belief that efficacy claims on WLS labels are accurate and the assumption that the natural origins of herbal ingredients are synonymous with them being safe. Despite their widespread use, WLS typically undergo less stringent regulation than conventional medicines and there is limited understanding of the safety of these products and their common active ingredients [3, 4]. There have been increasing reports of adverse events associated with WLS in the literature, particularly pertaining to the liver due to its important role in the metabolism of xenobiotics [13, 14, 18, 22, 44]. At present, the phytochemicals responsible for WLS‐induced toxicity are typically either suspected but unconfirmed or otherwise remain unknown.

The current study assessed *in vitro* safety of 12 phytochemicals commonly found in WLS using HepG2 and Caco‐2 cells. Cytotoxicity screening was first performed to identify active compounds of concern, followed by untargeted ^1^H NMR spectroscopy to determine the metabolic changes induced by the selected phytochemicals. EGCG was the only compound that caused cytotoxicity in both cell lines, and metabolic profiling indicated changes in key metabolites such as amino acids (e.g., alanine, leucine, threonine, valine), carboxylic acids (e.g., acetate, citrate, lactate) and amines (e.g., glycerophosphocholine, phosphocholine).

Cell membranes, of which the major constituent is phosphatidylcholine, are commonly impacted during toxicity [45, 46]. The primary mechanism for cell membrane damage is lipid peroxidation, and this can lead to loss of membrane function and ultimately result in cell death [45, 47]. Phosphocholine concentrations were significantly altered in both cell lines along with glycerophosphocholine levels in Caco‐2 cells. These metabolites are degradation products of phosphatidylcholine; therefore, alterations in their concentration likely indicate lipid peroxidation in response to EGCG.

Both cell lines had an accumulation of proteinogenic amino acids such as glycine, isoleucine and valine, suggestive of damage to intracellular proteins [34, 35, 48, 49]. This is supported by elevated levels of methylmalonate in Caco‐2 cells, which is a by‐product of protein degradation [50]. In addition to the breakdown of proteins, changes in certain amino acids could be indicative of intracellular demand for alternative fuel sources [51, 52]. Alanine, glutamine, glutamate and serine can all be converted into pyruvate, and this can subsequently be utilised for oxidative phosphorylation, fermentation or gluconeogenesis depending on cellular capabilities [53]. Isoleucine was increased in both cell lines, and this could be in part due to its mobilisation for the production of succinyl‐CoA, a TCA cycle intermediate [53]. Further supporting the theory of a requirement for noncarbohydrate energy sources is the accumulation of glucose in both cell lines. This was coupled with a decrease in pyruvate in Caco‐2 cells, collectively suggesting a breakdown in glycolytic function in response to EGCG. Lactate levels were elevated in both cell lines, indicating increased activation of fermentation pathways potentially in response to reduced aerobic respiration.

In HepG2 cells, EGCG treatment caused disruption to the TCA cycle as indicated by an accumulation of intermediates citrate and fumarate. This accumulation of citrate likely inhibited phosphofructokinase activity in the glycolytic pathway, contributing to an overall reduction in glycolysis and increase in intracellular glucose [54]. EGCG‐treated Caco‐2 cells had a decrease in citrate but the TCA cycle appeared less perturbed with no significant alterations in succinate [55]. A 2018 study examined the effects of EGCG‐induced hepatotoxicity in mice and found it caused dysfunction in mitochondria due to mitochondrial membrane collapse, consistent with the changes in metabolic profile discussed in the HepG2 cells [56].

Lipid peroxidation, protein degradation and disruption to energy metabolism are all characteristics of oxidative stress [46, 48]. During normal cellular metabolism, free radicals such as reactive oxygen species (ROS) are produced in small amounts [46, 48]. Endogenous antioxidant mechanisms quickly neutralise these products before they can cause damage within cells [57, 58]. When free radical production outstrips the antioxidant capacity of cells, it results in a pro‐oxidant state within cells. This allows free radicals to persist and cause damage to important cellular components such as proteins and lipids. Initially, homeostatic mechanisms within cells are able to adapt and combat oxidative stress to prolong survival; however, once these processes are overwhelmed, cell death can occur via apoptosis or necrosis [47, 59]. In this study, the observed decrease in cell viability with increasing EGCG concentration reflects both cell lines succumbing to oxidative damage.

**Fig. 4:**
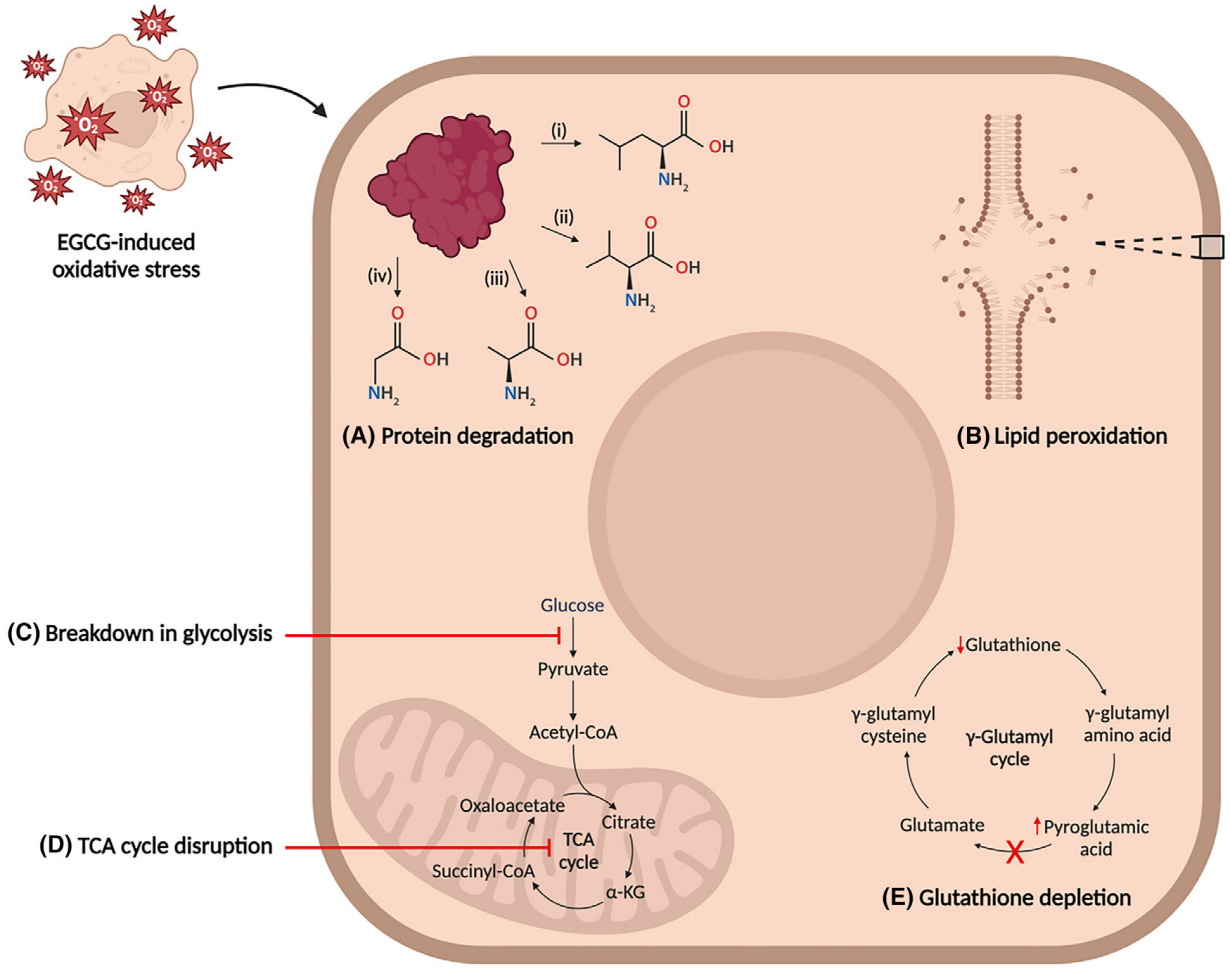
Summary of key intracellular mechanisms disrupted in HepG2 and Caco‐2 cells in response to EGCG, including (A) protein degradation resulting in the accumulation of amino acids such as (i) leucine, (ii) valine, (iii) alanine and (iv) glycine; (B) lipid peroxidation; (C, D) disrupted carbohydrate metabolism; and (E) glutathione depletion. Created with BioRender.com.

Glutathione is the dominant antioxidant within cells and is produced via the γ‐glutamyl cycle [60, 61]. It is capable of directly detoxifying free radicals by donating electrons, or indirectly by acting as a cofactor for antioxidant enzymes such as glutathione peroxidase [57, 60]. Glutathione levels were decreased although not significantly in Caco‐2 cells treated with EGCG, which could be due to oxidative stress. Pyroglutamate, an intermediate in the γ‐glutamyl cycle, was significantly increased in treated Caco‐2 cells, which may be due to increased cellular demand for glutathione in response to EGCG [61, 62]. Accumulation of pyroglutamate in the presence of decreased glutathione levels potentially reflects a perturbation in the γ‐glutamyl cycle. It should be noted that neither glutathione nor pyroglutamate could be measured in the HepG2 cells indicating the metabolite peaks were too small to get accurate integrals. A summary of the biochemical changes observed in response to EGCG toxicity is depicted in Fig. 4.

EGCG is the most abundant catechin in *C. sinensis* and the toxicity observed in this study is consistent with increasing reports of adverse events associated with this herb, particularly in the form of green tea extract (GTE) [14, 56, 63, 64, 65]. GTE has been implicated in numerous cases of hepatotoxicity in recent years [14, 63, 65]. Cases of GTE‐induced liver damage have been observed in individuals consuming single‐ingredient products only containing *C. sinensis* and in those consuming GTE as part of a multi‐ingredient supplement. In many instances, it has been confirmed as the hepatotoxic agent following the observation that discontinuation of GTE led to resolution of liver injury [66, 67]. Onset of GTE‐induced liver damage is often acute with a hepatocellular pattern of injury, and in some cases, this has been so severe that transplantation was necessary for patient survival [14, 68, 69, 70].

An increase in cell viability was observed in HepG2 cells treated with raspberry ketone, Caco‐2 cells exposed to epigallocatechin, chlorogenic acid and hydroxycitric acid lactone, and both cell lines in response to epicatechin, catechin hydrate, synephrine, capsaicin, forskolin, caffeine and hydroxycitric acid tripotassium salt. This may occur in supplements containing these compounds, with a 2025 study showing a similar increase in HepG2 cell viability in response to a single‐ingredient *G. gummi‐gutta* WLS as was observed for hydroxycitric acid tripotassium salt in this cell line in the current study [71]. Given that HepG2 and Caco‐2 cells are cancer cell lines, this increase in cell viability may indicate a potential for WLS containing these compounds to cause increased proliferation of cancerous cells in humans. Metabolomic elucidation of whether these phytochemicals elicit metabolite changes consistent with biosynthetic pathways involved in cell proliferation would make a valuable contribution to current understanding of potential *in vivo* effects of these compounds.

Limitations of this study include the use of immortalised cell lines and higher phytochemical concentrations than typically expected *in vivo*, along with the analysis of phytochemicals in isolation when even single‐ingredient WLS usually comprise multiple active compounds. Immortalised cell lines such as the HepG2 and Caco‐2 cells used in this study are frequently used to model cytotoxicity [72, 73, 74, 75], with benefits including their genetic homogeneity, ease of maintenance, cost‐effectiveness and avoidance of the Hayflick limit facing primary cells [76, 77]. Conversely, due to the genetic changes required for these cell lines to replicate indefinitely, future studies should include the use of primary cell lines to more accurately reflect *in vivo* responses. Furthermore, this study pretreated HepG2 and Caco‐2 cells with rifampicin as metabolism in these cell lines is known to be reduced compared to physiological activity *in vivo* [37, 39, 78]. Rifampicin has been shown to induce CYP450 activity in immortalised cells and may also induce enzymes involved in Phase II metabolism such as UDP‐glucuronosyltransferases and sulfonyltransferases [39, 79, 80]. Rifampicin induction is commonly used in cytotoxicity studies to enhance metabolic activity in immortalised cells, [72, 74] though differences likely remain compared to normal *in vivo* cell function.

Phytochemical concentrations included in this study likely do not reflect *in vivo* concentrations and were based on similar studies evaluating the effects of herbal active compounds on human cells [33, 34, 36]. It is challenging to determine the typical daily intake of phytochemicals from WLS, as concentrations of active compounds differ between products due to variation in composition and directions for use. It is also difficult to determine frequency and duration of WLS use as access to these products is typically unsupervised. A 2025 review reported physiological EGCG serum concentrations as being 1–10 μm [81], however serum measurements reflect what remains intact following first‐pass metabolism, and therefore the gastrointestinal tract is likely exposed to higher concentrations. There are limited data for the physiological concentrations of other phytochemicals used in this study. Future research should include an evaluation of physiological concentrations in the gastrointestinal tract, in addition to serum concentrations of active phytochemical metabolites.

Although the remaining 11 phytochemicals analysed did not elicit toxicity, this only indicates their safety in isolation at the doses tested in this study. WLS rarely contain these compounds in isolation, rather, they are included as part of a complex chemical mixture [22, 82]. Evaluating a single compound in isolation as was done in this study may not accurately represent the overall effects of WLS containing that phytochemical. It is important to understand how the phytochemicals in each herb interact to establish their safety both in isolation and in combination with other herbal constituents in multi‐ingredient supplements. Current knowledge of phytochemical‐phytochemical and phytochemical‐drug interactions is limited to reports in the literature following adverse events in consumers. For example, *G. gummi‐gutta* has been implicated in cases of neurotoxicity, specifically episodes of mania, when taken in combination with selective serotonin reuptake inhibitors (SSRIs) [17, 31]. In a 2014 case, this neurotoxicity subsequently resolved upon cessation of SSRI treatment [17]. The major active compound in *Garcinia* supplements is HCA, which was examined in two forms in this study. The mechanism of neurotoxicity is thought to be due to the serotonergic activity of HCA leading to synergism between this phytochemical and the SSRI prescribed resulting in serotonin toxicity [17]. Given the serious nature of adverse events relating to interactions between herbal supplements and other medicinal products, it is important that future research focusses on the elucidation of other potentially harmful interactions associated with these products.

## Conclusion

This study assessed the safety of 12 phytochemicals commonly found in WLS using an *in vitro* model with HepG2 and Caco‐2 cells. EGCG was the only compound that caused a significant reduction in cell viability, and a shift in the metabolomic profile of the cells. This suggests that the inclusion of EGCG in WLS should be more stringently regulated to protect consumers of these products. While the remaining 11 phytochemicals analysed were not observed to cause toxicity in isolation, future research should focus on the interactions between these active compounds and other substances included in WLS before their general safety can be assured. Until the biological activity of these compounds and their potential interactions are fully understood, improved independent premarket safety analysis of WLS entering the market and increasing education among medical professionals in relation to the potential for drug‐herb interactions is necessary to ensure consumer well‐being.

## Conflict of interest

The authors declare no conflict of interest.

## Author contributions

ECD performed the experiments, performed data analysis, prepared visualisations and wrote the original draft. GLM conceived and designed the project, contributed to methodology development for cell work and supervised the study. IFM contributed to methodology development for cell work and supervised the study. SLL contributed to methodology development for NMR analyses, curated the NMR data, supervised the study and prepared visualisations. All authors read, edited and approved the manuscript.

## Supporting information


**Fig. S1.** HepG2 and Caco‐2 cell viability following 48 h treatment with epigallocatechin.
**Fig. S2.** HepG2 and Caco‐2 cell viability following 48 h treatment with epicatechin.
**Fig. S3.** HepG2 and Caco‐2 cell viability following 48 h treatment with catechin hydrate.
**Fig. S4.** HepG2 and Caco‐2 cell viability following 48 h treatment with chlorogenic acid.
**Fig. S5.** HepG2 and Caco‐2 cell viability following 48 h treatment with synephrine.
**Fig. S6.** HepG2 and Caco‐2 cell viability following 48 h treatment with raspberry ketone.
**Fig. S7.** HepG2 and Caco‐2 cell viability following 48 h treatment with capsaicin.
**Fig. S8.** HepG2 and Caco‐2 cell viability following 48 h treatment with forskolin.
**Fig. S9.** HepG2 and Caco‐2 cell viability following 48 h treatment with caffeine.
**Fig. S10.** HepG2 and Caco‐2 cell viability following 48 h treatment with HCA‐lactone.
**Fig. S11.** HepG2 and Caco‐2 cell viability following 48 h treatment with HCA‐potassium salt.

## Data Availability

The data that support the findings of this study are available at DOI: 10.5281/zenodo.17595924.
