# Supplementary material for: Evaluation of in vitro toxicity of common phytochemicals included in weight loss supplements using 1H NMR spectroscopy
Source: FEBS Open Bio. 2025 Dec 9;16(5):906–20. doi: 10.1002/2211-5463.70170 (PMC13145343; doi:10.1002/2211-5463.70170)
Supplement: Supplementary file 1 — Fig. S1. HepG2 and Caco‐2 cell viability following 48 h treatment with epigallocatechin. Fig. S2. HepG2 and Caco‐2 cell viability following 48 h treatment with epicatechin. Fig. S3. HepG2 and Caco‐2 cell viability following 48 h treatment with catechin hydrate. Fig. S4. HepG2 and Caco‐2 cell viability following 48 h treatment with chlorogenic acid. Fig. S5. HepG2 and Caco‐2 cell viability following 48 h treatment with synephrine. Fig. S6. HepG2 and Caco‐2 cell viability following 48 h treatment with raspberry ketone. Fig. S7. HepG2 and Caco‐2 cell viability following 48 h treatment with capsaicin. Fig. S8. HepG2 and Caco‐2 cell viability following 48 h treatment with forskolin. Fig. S9. HepG2 and Caco‐2 cell viability following 48 h treatment with caffeine. Fig. S10. HepG2 and Caco‐2 cell viability following 48 h treatment with HCA‐lactone. Fig. S11. HepG2 and Caco‐2 cell viability following 48 h treatment with HCA‐potassium salt. [file FEB4-16-906-s001.pdf]

Evaluation of *in vitro* toxicity of common phytochemicals included in weight loss supplements using ^1H NMR spectroscopy

Emily C. Davies[1,2], Garth L. Maker[1,2], Ian F. Musgrave[3], Samantha Lodge[1]

[1]Centre for Computational and Systems Medicine, Murdoch University, Perth, WA 6150, Australia

[2]Medical, Molecular and Forensic Sciences, Murdoch University, 90 South Street, Murdoch, WA 6150, Australia

[3]Adelaide Medical School, The University of Adelaide, Adelaide, SA 5005, Australia

Correspondence to:
Samantha Lodge: sam.lodge@murdoch.edu.au
Garth Maker G.Maker@murdoch.edu.au

Key words: herbal weight loss supplements, ^1H NMR spectroscopy, HepG2 cells, Caco-2 Cell, metabolomics, oxidative stress, epigallocatechin-3,0-gallate, EGCG.

[OFFICIAL]

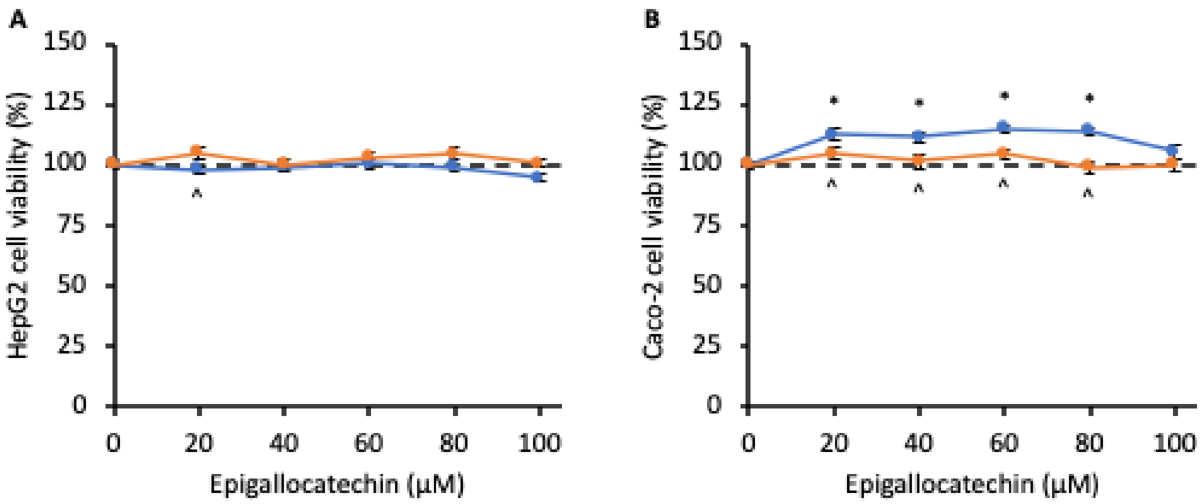

Figure S1. HepG2 (left) and Caco-2 (right) cell viability following 48 h treatment with epigallocatechin (n=15). Orange: cells pre-treated with rifampicin to induce CYP450 activity; blue: cells did not undergo pre-treatment. * Indicates concentrations where cell viability is significantly different from cells not treated with EGCG, in cells that did not undergo pre-treatment with rifampicin. ^ denotes a significant difference between rifampicin-induced and non-induced cells. MTT data were first investigated using a one-way analysis of variance (ANOVA) to determine the effects of WLS compared to the control. This was followed by a Dunnett's post hoc test to determine p-values at each concentration compared to control, with a significance of $p<0.01$ selected for all experiments. Error bars indicate SEM.

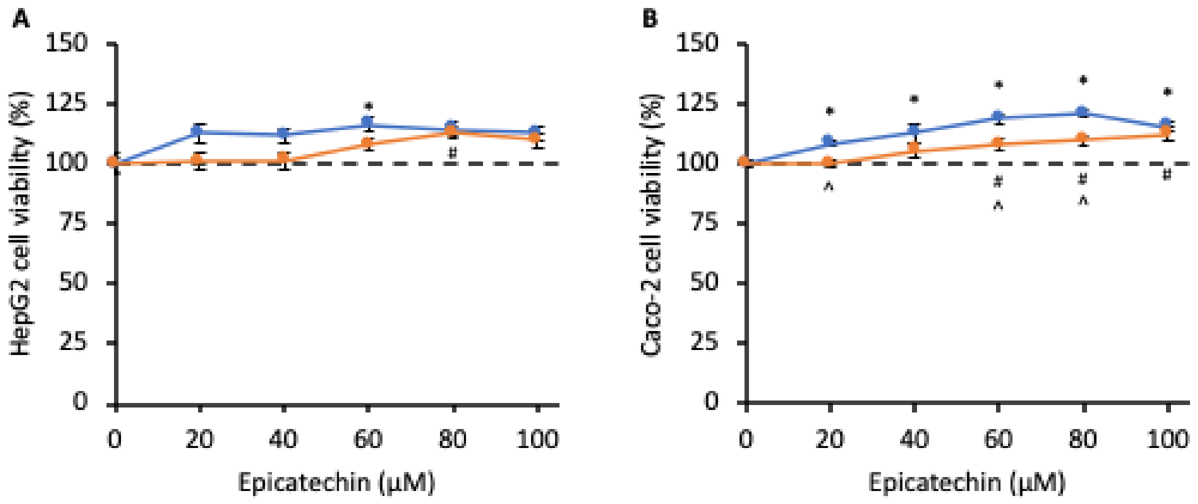

Figure S2. HepG2 (left) and Caco-2 (right) cell viability following 48 h treatment with epicatechin (n=15). Orange: cells pre-treated with rifampicin to induce CYP450 activity; blue: cells did not undergo pre-treatment. * Indicates concentrations where cell viability is

significantly different from cells not treated with EGCG, in cells that did not undergo pre-treatment with rifampicin. ^ denotes a significant difference between rifampicin-induced and non-induced cells. MTT data were first investigated using a one-way analysis of variance (ANOVA) to determine the effects of WLS compared to the control. This was followed by a Dunnett's post hoc test to determine p-values at each concentration compared to control, with a significance of p<0.01 selected for all experiments. Error bars indicate SEM.

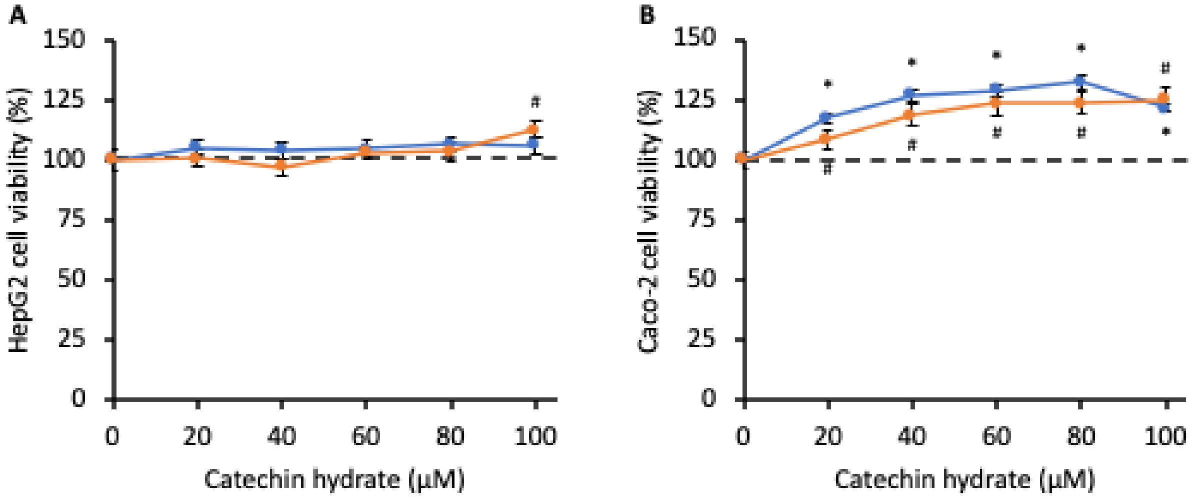

Figure S3. HepG2 (left) and Caco-2 (right) cell viability following 48 h treatment with catechin hydrate (n=15). Orange: cells pre-treated with rifampicin to induce CYP450 activity; blue: cells did not undergo pre-treatment. */# indicate concentrations where cell viability is significantly different from cells not treated with EGCG. MTT data were first investigated using a one-way analysis of variance (ANOVA) to determine the effects of WLS compared to the control. This was followed by a Dunnett's post hoc test to determine p-values at each concentration compared to control, with a significance of p<0.01 selected for all experiments. Error bars indicate SEM.

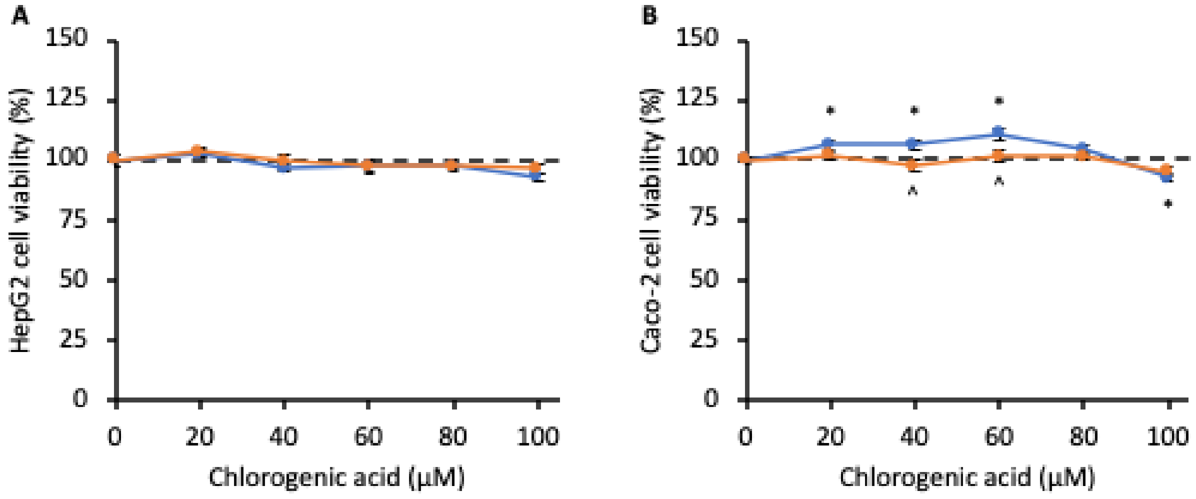

[OFFICIAL]

Figure S4. HepG2 (left) and Caco-2 (right) cell viability following 48 h treatment with chlorogenic acid (n=15). Orange: cells pre-treated with rifampicin to induce CYP450 activity; blue: cells did not undergo pre-treatment. * Indicates concentrations where cell viability is significantly different from cells not treated with EGCG, in cells that did not undergo pre-treatment with rifampicin. ^ denotes a significant difference between rifampicin-induced and non-induced cells. MTT data were first investigated using a one-way analysis of variance (ANOVA) to determine the effects of WLS compared to the control. This was followed by a Dunnett's post hoc test to determine p-values at each concentration compared to control, with a significance of p<0.01 selected for all experiments. Error bars indicate SEM.

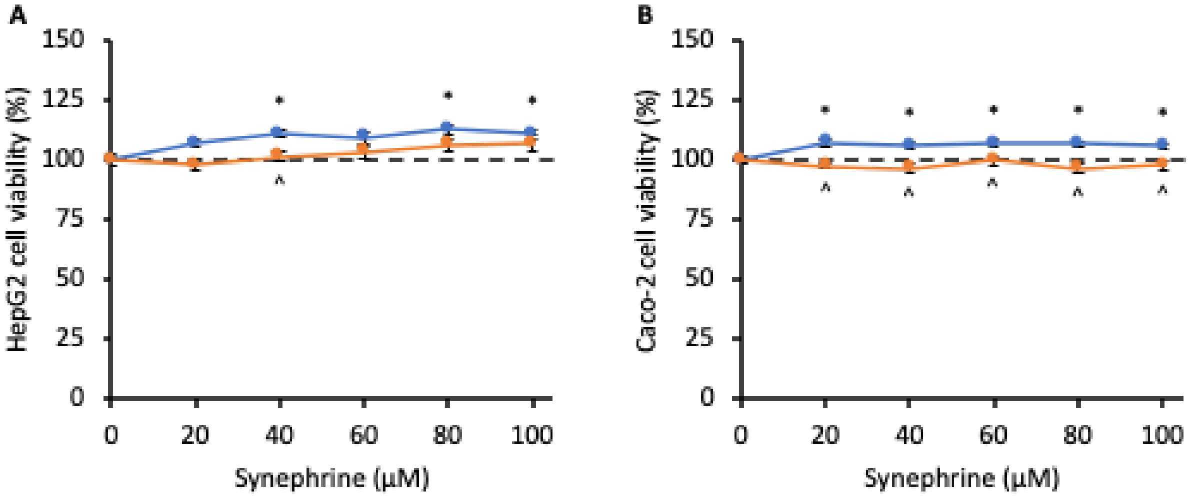

Figure S5. HepG2 (left) and Caco-2 (right) cell viability following 48 h treatment with synephrine (n=15). Orange: cells pre-treated with rifampicin to induce CYP450 activity; blue: cells did not undergo pre-treatment. * indicates concentrations where cell viability is significantly different from cells not treated with EGCG, in cells that did not undergo pre-treatment with rifampicin. ^ denotes a significant difference between rifampicin-induced and non-induced cells. MTT data were first investigated using a one-way analysis of variance (ANOVA) to determine the effects of WLS compared to the control. This was followed by a Dunnett's post hoc test to determine p-values at each concentration compared to control, with a significance of p<0.01 selected for all experiments. Error bars indicate SEM.

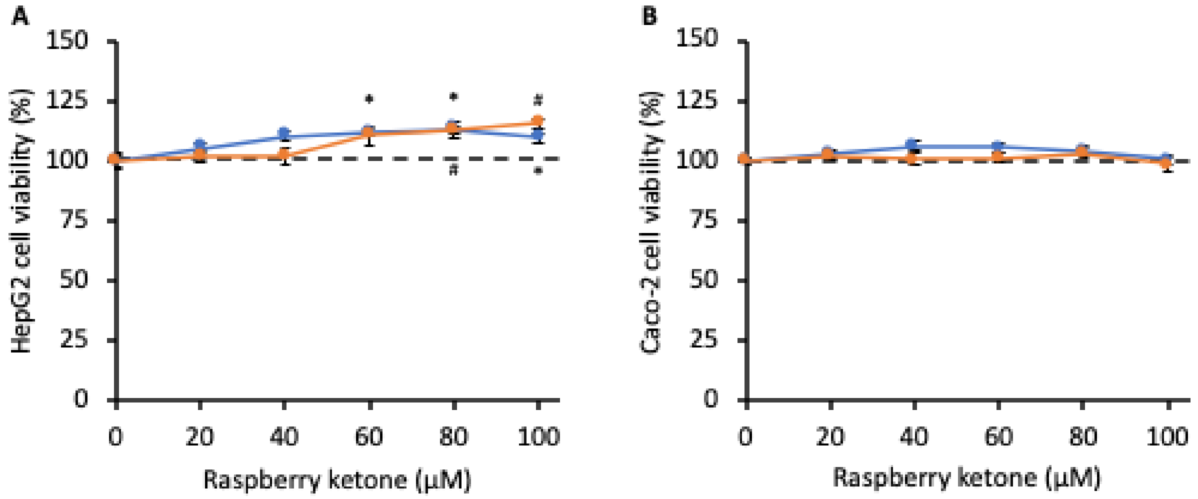

Figure S6. HepG2 (left) and Caco-2 (right) cell viability following 48 h treatment with raspberry ketone (n=15). Orange: cells pre-treated with rifampicin to induce CYP450 activity; blue: cells did not undergo pre-treatment. */# indicate concentrations where cell viability is significantly different from cells not treated with EGCG. MTT data were first investigated using a one-way analysis of variance (ANOVA) to determine the effects of WLS compared to the control. This was followed by a Dunnett's post hoc test to determine p-values at each concentration compared to control, with a significance of $p<0.01$ selected for all experiments. Error bars indicate SEM.

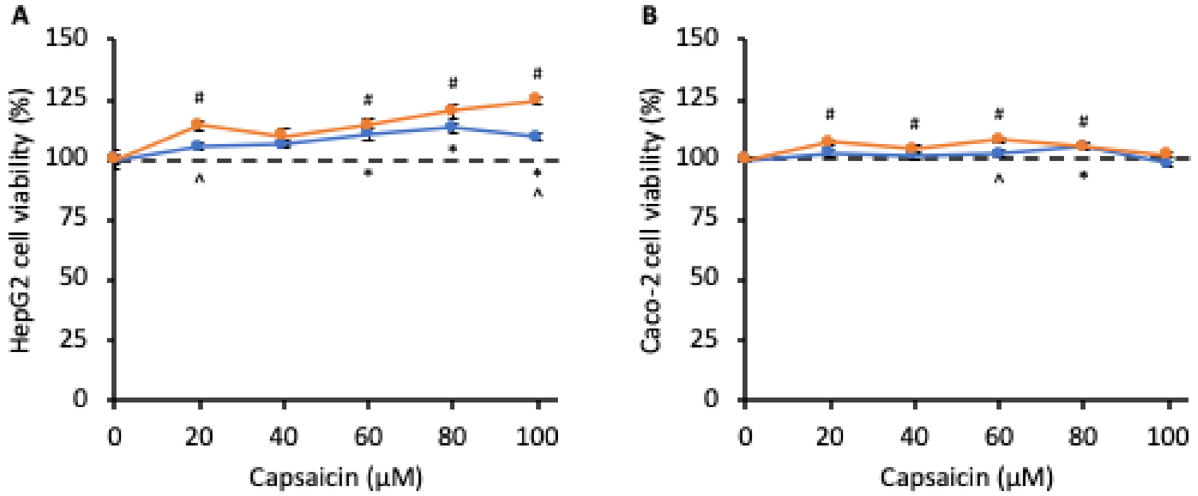

Figure S7. HepG2 (left) and Caco-2 (right) cell viability following 48 h treatment with capsaicin (n=15). Orange: cells pre-treated with rifampicin to induce CYP450 activity; blue: cells did not undergo pre-treatment. */# indicate concentrations where cell viability is significantly different from cells not treated with EGCG. ^ denotes a significant difference between rifampicin-induced and non-induced cells. MTT data were first investigated using a one-way analysis of variance (ANOVA) to determine the effects of WLS compared to the control. This was followed by a Dunnett's post hoc test to determine p-values at each

[OFFICIAL]

concentration compared to control, with a significance of p<0.01 selected for all experiments. Error bars indicate SEM.

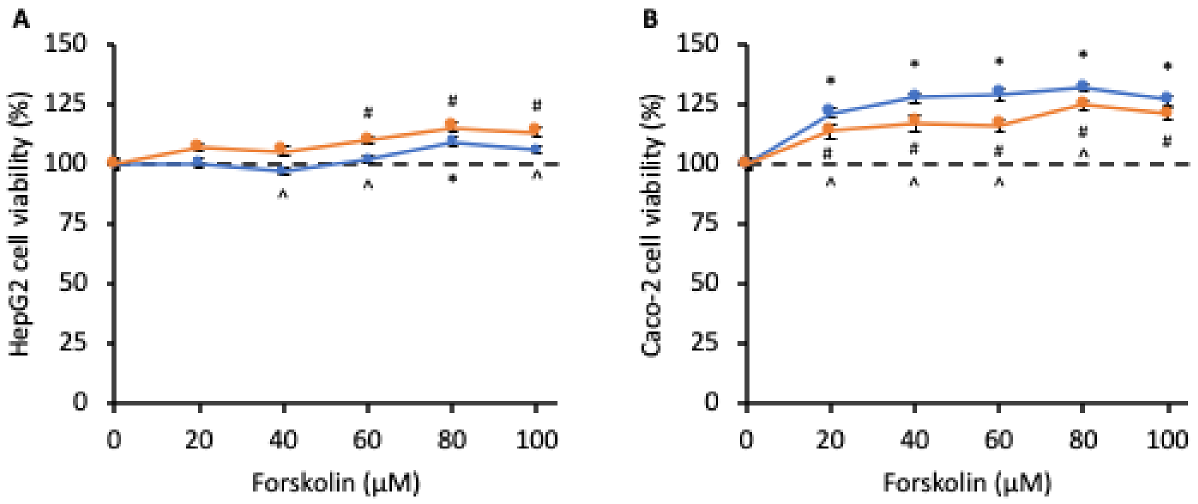

Figure S8. HepG2 (left) and Caco-2 (right) cell viability following 48 h treatment with forskolin (n=15). Orange: cells pre-treated with rifampicin to induce CYP450 activity; blue: cells did not undergo pre-treatment. */# indicate concentrations where cell viability is significantly different from cells not treated with EGCG. ^ denotes a significant difference between rifampicin-induced and non-induced cells. MTT data were first investigated using a one-way analysis of variance (ANOVA) to determine the effects of WLS compared to the control. This was followed by a Dunnett's post hoc test to determine p-values at each concentration compared to control, with a significance of p<0.01 selected for all experiments. Error bars indicate SEM.

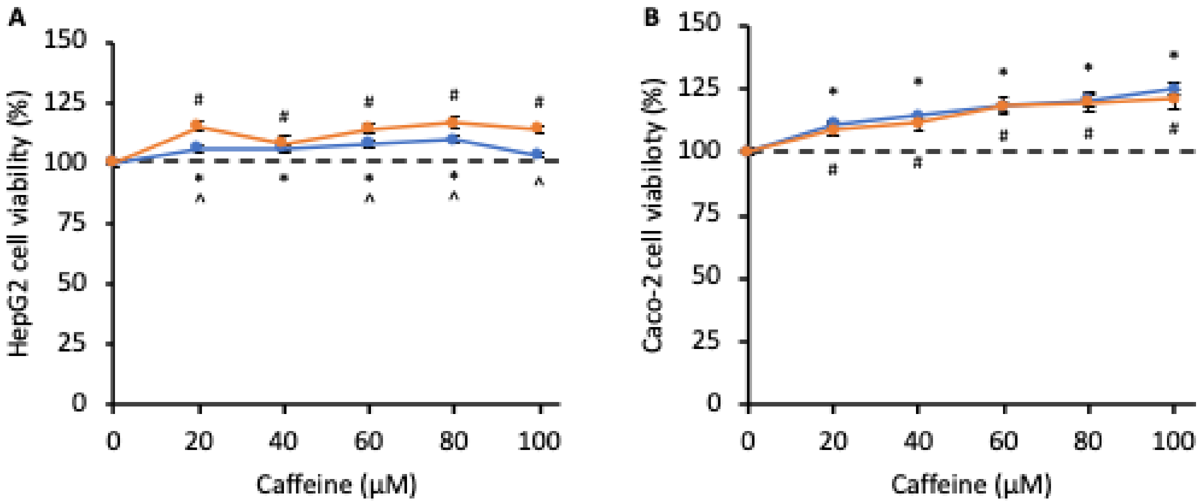

Figure S9. HepG2 (left) and Caco-2 (right) cell viability following 48 h treatment with caffeine (n=15). Orange: cells pre-treated with rifampicin to induce CYP450 activity; blue: cells did not

[OFFICIAL]

undergo pre-treatment. */# indicate concentrations where cell viability is significantly different from cells not treated with EGCG. ^ denotes a significant difference between rifampicin-induced and non-induced cells. MTT data were first investigated using a one-way analysis of variance (ANOVA) to determine the effects of WLS compared to the control. This was followed by a Dunnett's post hoc test to determine p-values at each concentration compared to control, with a significance of p<0.01 selected for all experiments. Error bars indicate SEM.

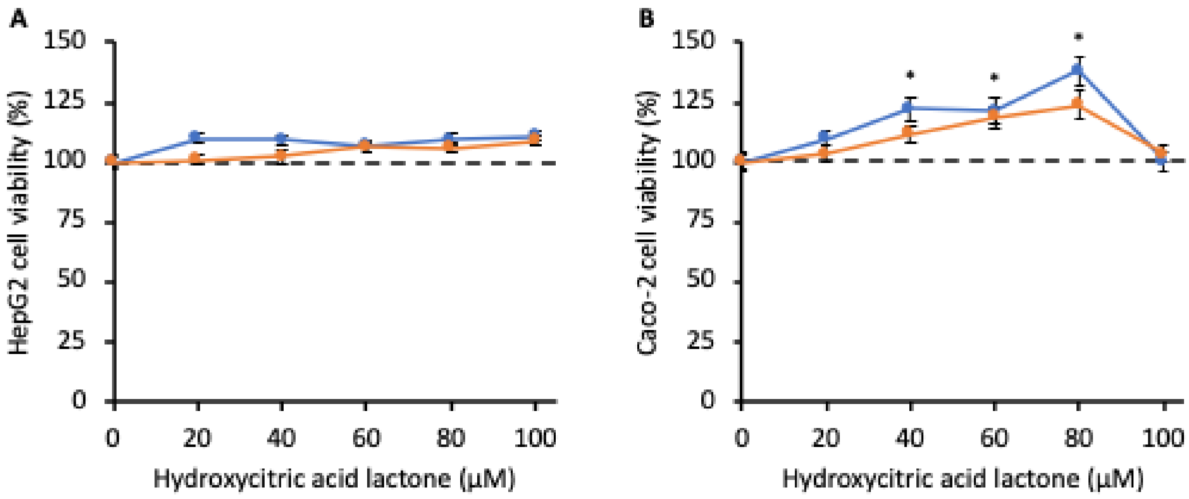

Figure S10. HepG2 (left) and Caco-2 (right) cell viability following 48 h treatment with HCA-lactone (n=15). Orange: cells pre-treated with rifampicin to induce CYP450 activity; blue: cells did not undergo pre-treatment. * indicates concentrations where cell viability is significantly different from cells not treated with EGCG, in cells that did not undergo pre-treatment with rifampicin. MTT data were first investigated using a one-way analysis of variance (ANOVA) to determine the effects of WLS compared to the control. This was followed by a Dunnett's post hoc test to determine p-values at each concentration compared to control, with a significance of p<0.01 selected for all experiments. Error bars indicate SEM.

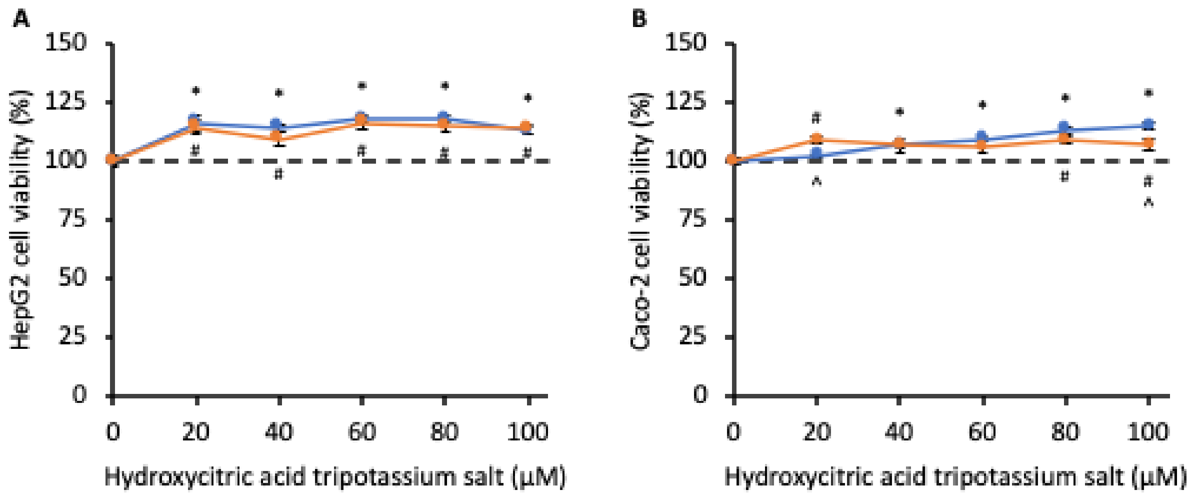

Figure S11. HepG2 (left) and Caco-2 (right) cell viability following 48 h treatment with HCA-potassium salt (n=15). Orange: cells pre-treated with rifampicin to induce CYP450 activity; blue: cells did not undergo pre-treatment. */# indicate concentrations where cell viability is significantly different from cells not treated with EGCG. ^ denotes a significant difference between rifampicin-induced and non-induced cells. MTT data were first investigated using a one-way analysis of variance (ANOVA) to determine the effects of WLS compared to the control. This was followed by a Dunnett's post hoc test to determine p-values at each concentration compared to control. Error bars indicate SEM.
